# Leukocyte counts and lymphocyte subsets in relation to pregnancy and HIV infection in Malawian women

**DOI:** 10.1111/aji.12678

**Published:** 2017-04-06

**Authors:** Wilson L. Mandala, Esther N. Gondwe, Malcolm E. Molyneux, Jenny M. MacLennan, Calman A. MacLennan

**Affiliations:** ^1^ Malawi‐Liverpool Wellcome Trust Clinical Research Programme College of Medicine Blantyre Malawi; ^2^ Department of Basic Medical Sciences College of Medicine Blantyre Malawi; ^3^ Liverpool School of Tropical Medicine Liverpool UK; ^4^ Department of Zoology University of Oxford Oxford UK; ^5^ The Jenner Institute Nuffield Department of Medicine University of Oxford Oxford UK; ^6^ Institute of Immunology and Immunotherapy College of Medicine and Dental Sciences University of Birmingham Birmingham UK

**Keywords:** HIV, leukocytes, lymphocytes, parturition, pregnancy, sepsis

## Abstract

**Problem:**

We investigated leukocyte and lymphocyte subsets in HIV‐infected or HIV‐uninfected, pregnant or non‐pregnant Malawian women to explore whether HIV infection and pregnancy may act synergistically to impair cellular immunity.

**Method of study:**

We recruited 54 pregnant and 48 non‐pregnant HIV‐uninfected women and 24 pregnant and 20 non‐pregnant HIV‐infected Malawian women. We compared peripheral blood leukocyte and lymphocyte subsets between women in the four groups.

**Results:**

Parturient HIV‐infected and HIV‐uninfected women had more neutrophils (each *P*<.0001), but fewer lymphocytes (*P*<.0001; *P*=.0014) than non‐pregnant women. Both groups had fewer total T cells (*P*<.0001; *P*=.002) and CD8^+^ T cells (*P*<.0001; *P*=.014) than non‐pregnant women. HIV‐uninfected parturient women had fewer CD4^+^ and γδ T cells, B and NK cells (each *P*<.0001) than non‐pregnant women. Lymphocyte subset percentages were not affected by pregnancy.

**Conclusion:**

Malawian women at parturition have an increased total white cell count due to neutrophilia and an HIV‐unrelated pan‐lymphopenia.

## 1 Introduction

In the Global Burden of Disease 2010 study, 254 700 deaths were attributed to maternal conditions, accounting for 7.3% of global deaths in women aged 15 to 49 years. Of these, 21 900 were estimated to be due to maternal sepsis^1^ and recent reports indicate that as many as 11% of global maternal mortality is due to sepsis.^2^ A focused analysis of maternal deaths in 2011 estimated that 99% (270 772/273 465) occurred in the developing world with 52% in sub‐Saharan Africa.^3^ Millennium Development Goal number 5 was aimed at lowering the maternal mortality ratio (MMR), the number of maternal deaths per 100 000 live births, by three‐quarters from 1990 to 2015,^4^ yet, as of 2015, only 20 countries had achieved or were regarded to be on track to achieving this goal.^2^

There has been a growing appreciation of the impact of the HIV epidemic in hindering progress towards achieving Millennium Development Goal number 5. From 1980 to 1990, global maternal mortality was decreasing by 1.8% per year. With the escalation of the HIV/AIDS pandemic since the early 1990s, this decline slowed to 1.4% per year for the period 1990‐2008.^2^ From 1990 to 2000, the maternal mortality ratio (MMR) increased in many countries in sub‐Saharan Africa with large HIV burdens; in Malawi, the reported MMR doubled in that decade from 606 to 1397 per 100 000 live births.^3^ With the implementation of antiretroviral programmes, this trend has been reversed, and in 2011, the MMR in Malawi had fallen to 422.^3^ Nevertheless, despite availability of ART, 20.5% (56 100) of maternal deaths worldwide in 2010 were HIV‐related.^1^

In South Africa, nationwide confidential enquiries into facility‐based deaths among pregnant women found that the MMR was almost 10‐fold higher among HIV‐infected women (328/100 000) than non‐infected women (34/100 000).^5^ A key question pertains to the underlying cause of these HIV‐related maternal deaths. Sepsis and other infections secondary to compromised immunity could be responsible.^6^ In the South African series, 53% (2102/3959) of maternal deaths were due to infection, with 28% of these attributed to pregnancy‐related sepsis, often post‐partum, and 72% to non‐pregnancy‐related infections such as pneumonia and tuberculosis. Overall, 90% of the pregnancy‐related sepsis and 96% of those non‐pregnancy‐related infections occurred in HIV‐infected individuals.^5^

Pregnancy itself is a well‐recognized cause of altered immunity, particularly cellular immunity^7^ affecting CD4^+^ T lymphocytes,^8^ the same cells that are targeted by HIV. We therefore hypothesized that HIV infection and pregnancy may act synergistically to impair cellular immunity, reducing CD4 counts and potentially increasing susceptibility to infection. To explore this possibility among African women, we performed a prospective cross‐sectional study of HIV‐infected and HIV‐uninfected pregnant and non‐pregnant Malawians attending a health centre in Blantyre and compared leukocyte and lymphocyte subsets at parturition with those in non‐pregnant women.

## 2 Materials and Methods

### 2.1 Study area and population

We recruited pregnant and non‐pregnant women between 26 September 2006 and 15 January 2007 from Ndirande Health Centre (NHC) in Blantyre, Malawi, as they attended routine health checks or came for antenatal clinics. Informed consent was obtained from each participant. Exclusion criteria were presence of active disease and/or fever and receiving medication at the time of recruitment. Blood samples were tested for HIV infection and malaria parasitaemia. Data from participants diagnosed with malaria were excluded from final statistical analysis. A 5‐mL venous blood aliquot was taken from each participant, on the day of delivery from parturient women or on the day of recruitment from non‐pregnant women. Seven of the 20 HIV‐infected non‐pregnant women were aged between 60 and 68, which is above the child‐bearing age and were included in the analysis to increase the sample size for this group. All HIV‐infected non‐pregnant women were not aware of their HIV status prior to being recruited into the study, and thus, none were receiving HAART. However, all HIV‐infected parturient women were taking nevirapine for different durations during their pregnancies.

### 2.2 Investigations

HIV testing was performed using two rapid test kits; Determine (Abbott Laboratories, Japan) and Unigold (Trinity Brotch, Dublin). Thick and thin blood smears on slides were prepared by standard methods for *Plasmodium falciparum* parasite detection. Total white cell count (WCC) and percentages and absolute counts of neutrophils, lymphocytes and monocytes were determined using a HMX Haematological Analyser (Coulter, USA).

### 2.3 Immunophenotyping

Immunophenotyping of blood samples by flow cytometry and lymphocyte subset identification was performed according to Table S1. In brief, 25 μL of EDTA blood was mixed with 1 μL of each antibody and incubated in the dark at room temperature for 15 minutes. About 500 μL of 1× FACS lysing solution (Becton Dickinson) was added to each tube and incubated in the dark for 10 minutes to lyse erythrocytes. Cells were washed twice with PBS and fixed with 1% formaldehyde (Sigma)/PBS and data acquired on a FACSCalibur Flow Cytometer. CellQuest was used for analysis using the gating strategy in Fig. S5.

### 2.4 Ethical approval

Ethical approval for this study was obtained from College of Medicine Research and Ethics Committee (COMREC) in Blantyre, Malawi.

### 2.5 Statistical analysis

Participants were grouped according to their pregnancy and HIV status. The median and range were determined for age and absolute and percentage leukocyte and lymphocyte subset proportion in each group and are reported in the table. 10^th^ and 90th percentiles were determined for use in the Figures. The Kruskal‐Wallis equality‐of‐populations rank test was used to identify overall differences between groups for each subset. Pairwise differences between two groups were then determined using the two‐sample Wilcoxon rank‐sum (Mann‐Whitney) test. A *P* value of <.05 was considered statistically significant at 95% level of confidence. All statistical analyses were performed using Stata version 14 (StataCorp 2015).

## 3 Results

### 3.1 Participants and global interaction of HIV and Pregnancy

We recruited 54 pregnant (median age 25 years [range: 17‐39]) and 48 non‐pregnant HIV‐uninfected women (median age: 22 [17‐31]) and 24 HIV‐infected pregnant women (median age: 25 [18‐37]) and 20 HIV‐infected non‐pregnant women (median age: 29 [18‐68]). Results of the global statistical analysis showed that with the exception of absolute monocyte counts and percentage of NK and γδ T cells, all other cell subsets were significantly different between groups (Table 1).

**Table 1:** Medians (range) of different leukocyte and lymphocyte subsets for the four different groups (HIV‐uninfected and non‐pregnant groups, HIV‐uninfected parturient, HIV‐infected and non‐pregnant and HIV‐infected parturient groups) presented either as percentages or absolute counts following a global statistical analysis of the data from all four groups

Group	HIV‐uninfected not pregnant	HIV‐uninfected parturient	HIV‐infected not pregnant	HIV‐infected parturient	Overall *P* value	*P* value	*P* value	*P* value	*P* value
	1	2	3	4		1 vs2	3 vs 4	1 vs 3	2 vs 4
Number of participants	48	54	20	24					
Median age (y, range)	21.6 (17‐31)	25.1 (17‐39)	29.3 (18‐68)	25.5 (17‐37)	<.001	<.001	.315	<.001	.536
Cell type									
Absolute counts (×1000)/μL									
WBC	5.8 (3.6‐13.8)	12.3 (3.9‐25.8)	4.5 (2.5‐6.8)	11.5 (6.8‐25.0)	.001	<.001	<.001	<.004	.286
Neutrophils	2.7 (1.5‐6.2)	10.3 (2.6‐23.3)	1.7 (0.9‐3.0)	8.7 (2.8‐22.8)	.001	<.001	<.001	<.001	.091
Lymphocytes	2.2 (1.4‐4.5)	1.2 (0.5‐2.3)	2.0 (1.1‐3.2)	1.5 (0.6‐2.3)	.001	<.001	.001	.165	.100
Monocytes	0.4 (0.2‐1.1)	0.6 (0.1‐2.1)	0.4 (0.3‐0.7)	0.5 (0.1‐6.2)	.472	.276	.256	.902	.862
Percentage									
Neutrophils	41.9 (21.8‐89.2)	67.0 (16.0‐95.0)	57.1 (26.4‐91.2)	74.7 (14.6‐91.6)	.001	<.001	.179	.014	.242
Lymphocytes	40.6 (7.2‐64.9)	23.4 (4.4‐61.6)	32.4 (5.9‐57.7)	15.5 (4.2 ‐52.4)	.006	<.001	.175	.141	.333
Monocytes	8.6 (1.9‐16.8)	5.8 (0.3‐38.1)	7.9 (0.9‐10.8)	5.0 (0.4‐29.7)	.002	.002	.253	.038	.439
T cells	71.8 (53.8‐91.1)	70.8 (51.7‐85.1)	78.5 (56.0‐95.1)	73.2 (51.8‐88.9)	.021	.920	.273	.005	.211
CD4+ T cells	41.7 (27.2‐57.2)	40.4 (26.2‐58.6)	17.6 (3.8‐39.4)	18.6 (6.8‐42.9)	.001	.904	.832	<.001	<.001
CD8+ T cells	27.3 (15.9‐69.93)	26.2 (11.1‐69.3)	55.1 (18.6‐88.8)	53.7 (15.6‐74.1)	.001	.445	.768	<.001	<.001
NK cells	11.2 (2.3‐33.9)	10.5 (1.6‐25.2)	7.5 (2.1‐25.0)	9.3 (4.6‐25.9)	.196	.646	.141	.053	.630
T cells	4.2 (0.8‐9.9)	3.8 (0.6‐10.8)	3.9 (1.3‐24.9)	3.6 (1.6‐15.1)	.257	.068	.580	.250	.360
B cells	10.1 (5.8‐21.8)	10.9 (2.3‐25.8)	6.3 (2.0‐15.2)	9.20 (4.4‐20.6)	<.001	.212	<.001	<.001	.051
Absolute counts/μL									
T cells	1544 (1011‐3363)	824 (422‐1731)	1568 (672‐2764)	1029 (404‐1904)	<.001	<.001	.002	.884	.087
CD4+ T cells	878 (629‐2037)	468 (248‐971)	359 (79‐1128)	297 (74‐786)	<.001	<.001	.120	<.001	<.001
CD8+ T cells	598 (270‐1509)	270 (137‐1039)	1047 (223‐2341)	654 (121‐1408)	<.001	<.001	.014	.002	<.001
NK cells	275 (41‐847)	125 (15‐379)	173 (33‐800)	153 (55‐414)	<.001	<.001	.549	.040	.603
T cells	102 (19‐302)	43 (4.1‐216)	65 (29‐696)	58 (15‐252)	<.001	<.001	.217	.038	.069
B cells	235 (102‐699)	134 (22‐567)	142 (43‐264)	124 (33‐256)	<.001	<.001	.846	<.001	.649

### 3.2 Among non‐pregnant women, HIV‐infected individuals have lower mean WCC, neutrophil and CD4^+^ cell counts and a higher mean CD8^+^ cell count than HIV‐uninfected individuals

To confirm the differences attributable to infection with HIV among Malawian women of child‐bearing age, we compared leukocyte and lymphocyte subset counts between HIV‐infected and HIV‐uninfected non‐pregnant Malawian women. Total WCC (*P*=.004), neutrophil counts (*P*<.001) (Table 1 and Fig. S1A) and CD4^+^ T‐cell counts (Fig. S1C) and percentages (Fig. S1D) (both *P*<.0001) were lower in HIV‐infected than HIV‐uninfected women, while CD8^+^ T‐cell counts (*P*=.002) (Fig. S1C) and percentages (*P*<.001) (Fig. S1D) and neutrophil percentages of total white cells (*P*=.014) (Fig. S1B, Table 1) were higher. B, NK and γδ T‐cell counts were also lower in the HIV‐infected group (Fig. S1D, Table 1).

### 3.3 For HIV‐uninfected Malawian women, those at parturition have a higher WCC and neutrophil count, but lower lymphocyte count, than non‐pregnant women

We explored the effect of pregnancy at parturition on leukocyte and lymphocyte subsets by comparing results between parturient and non‐pregnant HIV‐uninfected Malawian women. Parturient women had a higher median WCC than non‐pregnant women (*P*<.001) due to a neutrophilia, and approximately half as many lymphocytes as non‐pregnant women (*P*<.001) (Table 1 and Fig. S2A). As percentages of total WCC, parturient women had more neutrophils (*P*=.002) and fewer lymphocytes (*P*=.003) and fewer monocytes (*P*=.002) than non‐pregnant women (Fig. S2B). Parturient women had significantly smaller absolute numbers of lymphocyte subsets (total T, CD4^+^ T, CD8^+^ T, γδ T, B and NK cells) than non‐pregnant women (*P*<.001; Fig. S2C). In these HIV‐uninfected populations, parturient and non‐pregnant women had similar lymphocyte subset proportions (Fig S2D, Table 1).

### 3.4 Among pregnant women at parturition, HIV‐infected individuals have a lower CD4^+^ T‐cell count, and a higher CD8^+^ T‐cell count, than HIV‐uninfected women

**Figure 1:**
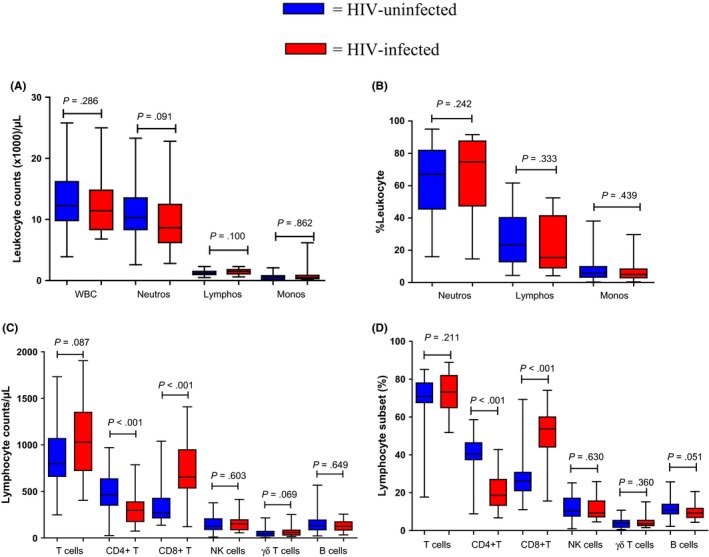
Medians for leukocyte subsets expressed as absolute counts (A) and as percentages (B) and lymphocyte subsets expressed as absolute counts (C) and as percentages (D) for HIV‐uninfected and pregnant women (blue boxes) and for HIV‐infected and parturient women (red boxes)

To investigate the effect of HIV infection on pregnancy, we compared the cell counts and proportions in pregnant HIV‐infected women with HIV‐uninfected pregnant women. Regardless of their HIV status, pregnant women had similar WBC, lymphocyte, monocyte and total T‐cell counts (Figure 1A,C and Table 1). As with non‐pregnant women, the HIV‐infected pregnant women had significantly (*P*<.001) fewer CD4^+^ T cells and more CD8^+^ T cells than HIV‐uninfected pregnant women. A similar picture was observed when these subsets were determined as a percentage of total lymphocytes (Figure 1D).

### 3.5 For HIV‐infected women, pregnant women have higher WCC and neutrophil counts, but lower lymphocyte counts compared to non‐pregnant women

We investigated the impact of pregnancy on Malawian women living with HIV, by comparing parturient HIV‐infected women with non‐pregnant HIV‐infected women. The effect of pregnancy on leukocyte and lymphocyte subsets was the same in HIV‐infected as HIV‐uninfected women.

HIV‐infected pregnant women had significantly (*P*<.001) higher WCC and neutrophil counts, but lower (*P*=.001) lymphocyte counts than non‐pregnant HIV‐infected women (Table 1 and Fig. S3A). Absolute counts of T cells and T‐cell subsets were reduced in HIV‐infected women, as in HIV‐uninfected women, but the reduction was less marked and the reduction in CD4^+^ T cells was not significant. Pregnant HIV‐infected women continued to have lower CD4^+^ T cells and higher CD8^+^ T cells than HIV‐uninfected women, both as absolute and percentage counts. There were no significant differences in CD4^+^ T cells, expressed either as a percentage or as absolute counts, between the two groups of HIV‐infected women. Overall, when expressed as a percentage, there were no differences between the pregnant and non‐pregnant women for all subsets except for B cells (*P*=.003) (Fig. S3D).

### 3.6 HIV‐infected and pregnant women have higher WCC and neutrophil counts, but lower mean lymphocyte, T‐cell, CD4+ T, NK and B‐cell counts compared to HIV‐uninfected and non‐pregnant women

Finally, to investigate the combined effect of simultaneous pregnancy and HIV infection, we compared the absolute cell counts and percentages in HIV‐infected parturient women with those in HIV‐uninfected non‐pregnant women. HIV‐infected pregnant women had higher (*P*<.001) WCC and neutrophil counts, but lower lymphocyte counts compared to HIV‐uninfected non‐pregnant women (Fig. S4A, Table 1). A similar picture was observed when these were presented as percentages (Fig. S4B). With the exception of CD8^+^ T‐cell counts, which did not differ between the two groups, HIV‐infected pregnant women had lower T cells, CD4^+^ T, NK and B‐cell counts compared to HIV‐uninfected non‐pregnant women (Fig. S4C). When presented as a percentage, HIV‐infected pregnant women had a higher proportion (*P*=.048) of CD8^+^ T cells compared to the HIV‐uninfected non‐pregnant women (Fig. S4D, Table 1).

## 4 Discussion

Our study firstly investigated only pregnancy‐associated changes in WCCs and lymphocyte subsets in HIV‐uninfected women and the effect of concurrent HIV infection and pregnancy on those changes. Previous studies^9^, ^10^, ^11^, ^12^, ^13^ had reported inconsistent results on how leukocyte and lymphocyte subsets vary in pregnancy of both HIV‐uninfected and HIV‐infected women in Africa. We observed that pregnancy alone, in the absence of HIV infection, was associated with a reduction in lymphocytes and T‐cell numbers across all subsets and with a neutrophilia consistent with what had been observed in some studies^14^, ^15^, ^16^, ^17^, ^18^ but not in others.^19^, ^20^, ^21^ This lack of consistence in changes in total lymphocytes and subsets in pregnancy found in the different studies could be because these cell subsets have previously been shown to be affected by other factors apart from pregnancy such as location and ethnic group.^22^

HIV infection is characterized by a reduction in total WCC, neutropenia and a particular reduction in CD4^+^ T cells but an increase in CD8^+^ T cells which is what we also observed in this study. Less well studied though is the effect of pregnancy on the leukocytes and lymphocyte subsets in HIV‐infected women. Contrary to our hypothesis, we found that the effect of pregnancy in HIV‐infected women was similar to that observed in HIV‐uninfected women effectively ruling out a possible synergistic effect of HIV infection and pregnancy as a possible factor for the increased proportion of death from sepsis in HIV‐infected pregnant women. Similar results were found in a study that recruited HIV‐infected pregnant women in United States of America which found that CD4^+^ T‐cell numbers were higher during pregnancy.^23^ Similarly, a study from Italy, in which pregnant and non‐pregnant HIV‐infected women were recruited and followed, only found that pregnant women had elevated CD4^+^ T‐cell counts at the point of recruitment.^24^ None of these two studies included HIV‐uninfected controls.

Among the few studies that have investigated leukocyte and lymphocyte subsets in pregnancy and HIV infection in the sub‐Saharan Africa (SSA), one recruited HIV‐infected and uninfected Malawian pregnant women in the third trimester and post‐partum but did not include non‐pregnant women.^25^ The study found that CD4^+^ and CD8^+^ absolute counts were higher post‐partum than in pregnancy, while CD4^+^ and CD8^+^ percentages did not change.^25^ Recent studies carried out in SSA^11^, ^12^, ^13^ have also reported that HIV infection has the same effect on leukocyte and lymphocyte subsets in pregnancy as it does in non‐pregnant women and does not seem to act synergistically to exacerbate the effect of pregnancy on these cell types. However, our study provides additional information because it included comparisons between the four different groups, HIV‐infected and pregnant, HIV‐infected non‐pregnant, HIV‐uninfected pregnant and HIV‐uninfected non‐pregnant and it investigated the variation in more cell types than the other previous studies conducted in SSA.^11^, ^12^, ^13^, ^25^ It is worth mentioning that CD4^+^ and CD8^+^ T cells have been observed to be stable when presented as a percentage of the total lymphocyte counts than when they are presented as totals counts^19^, ^23^ and this is similar to what we and others have reported before for HIV‐uninfected children during childhood.^26^, ^27^ Therefore, percentage CD4^+^ and CD8^+^ T‐cell values may have better application than absolute counts for assessing immunodeficiency during pregnancy with an added advantage that they can be more easily and accurately performed on flow cytometers.^28^, ^29^

There are several limitations to this work. Firstly, we only examined blood from pregnant women at the time of parturition but a longitudinal study tracking changes in individual women throughout pregnancy and post‐partum^9^ would provide a more comprehensive understanding of immunological changes in pregnancy. Secondly, although we investigated leukocyte and lymphocyte subset numbers and percentages, we did not examine their function and this could be crucial additional information. Thirdly, ideal comparison between the two HIV‐infected groups could have been achieved if they were known to be taking similar HAART^30^ and for the same period. Lastly, although the over‐aged (7/20) HIV‐infected women might not be considered representative controls for the HIV‐infected pregnant group, we had previously shown that the different leukocyte and lymphocyte subsets do not differ greatly between the ages of 18 years and beyond 60 years.^26^

## 5 Conclusion

We have examined the separate and combined effects of pregnancy and HIV infection on the cellular compartment of the immune system in Malawian women by measuring leukocyte and lymphocyte subsets. Although changes are seen that can be attributable to the interaction of both factors, our general finding is that the effect of pregnancy is the dominant factor for each individual immune parameter, apart from CD4^+^ and CD8^+^ T‐cell counts. Contrary to our initial speculation, pregnancy does not appear to exacerbate the CD4^+^ T‐cell lymphopenia of HIV infection, and therefore, simple immunophenotyping, such as that undertaken in this study, cannot easily explain the high levels of infection and mortality among pregnant women in SSA. Further studies are required to explore this and will ideally follow longitudinally a cohort of women of childbearing age and study cell percentage, numbers and function.

## Authors Contributions

WLM, JM and CAM conceived the study. WLM, JM and EG performed the investigations. JM and WM analysed the data. WLM, MEM, JM and CAM wrote the report. All authors contributed to the study design and reviewed the report.
