# Supplementary material for: Leukocyte counts and lymphocyte subsets in relation to pregnancy and HIV infection in Malawian women
Source: Am J Reprod Immunol. 2017 Apr 6;78(3):e12678. doi: 10.1111/aji.12678 (PMC5573949; doi:10.1111/aji.12678)
Supplement: Supplementary file 1 [file AJI-78-na-s001.doc]

**Figure S1:** Non-Pregnant Women; = HIV-uninfected

= HIV-infected

**Figure S2:** HIV-Uninfected Women; = Non-pregnant

= parturient women

**Figure S3:** HIV-Infected Women: = Non-pregnant

= Parturient women

**Figure S4:** HIV-Infected and Pregnant Women: = HIV-uninfected Non-pregnant women

= HIV-infected parturient women
