# Supplementary material for: Leukocyte counts and lymphocyte subsets in relation to pregnancy and HIV infection in Malawian women
Source: Am J Reprod Immunol. 2017 Apr 6;78(3):e12678. doi: 10.1111/aji.12678 (PMC5573949; doi:10.1111/aji.12678)
Supplement: Supplementary file 2 [file AJI-78-na-s002.doc]

**
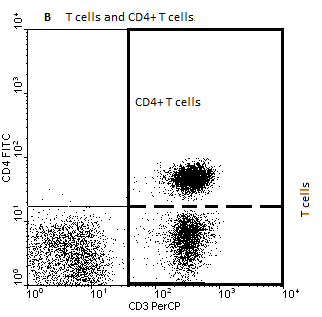

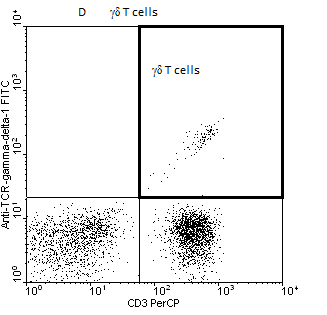

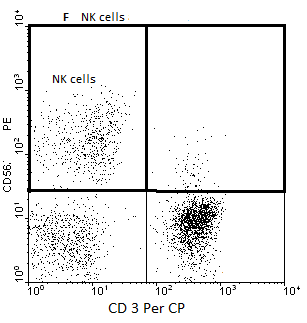
**

**
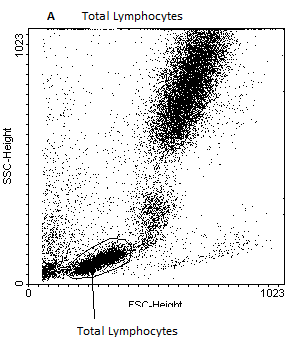

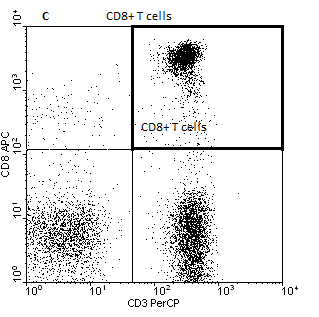

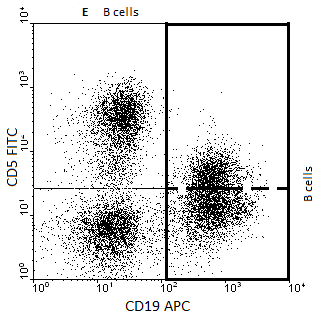
**

**Figure S1**: **Main** **Gating Strategy**: Flow cytometer dot plots illustrating the forward/side scatter plot (A) with *R1* gate for Total lymphocytes, total T cells and CD4+ T cells (B), CD8+ T cells (C), γδ T cells (D), B cells (E), and NK cells (F).
