# Supplementary material for: Leukocyte counts and lymphocyte subsets in relation to pregnancy and HIV infection in Malawian women
Source: Am J Reprod Immunol. 2017 Apr 6;78(3):e12678. doi: 10.1111/aji.12678 (PMC5573949; doi:10.1111/aji.12678)
Supplement: Supplementary file 3 [file AJI-78-na-s003.doc]

**Table S1**: Immunophenotyping strategy used in the study

| Tube No. | **Cell Type** | **Population** | **Monoclonal Antibodies** |
| --- | --- | --- | --- |
| 1 | T lymphocytes | CD3^+^ | Anti-CD3-PerCP |
| 2 | CD4+ T lymphocytes | CD3^+^CD4^+^ | Anti-CD3-PerCP,  Anti-CD4-FITC |
| 3 | CD8+ T lymphocytes | CD3^+^CD8^+^ | Anti-CD3-PerCP,  Anti-CD8-APC |
| 4 | B lymphocytes | CD19^+^ | Anti-CD19-APC |
| 7 | NK cells | CD3^-^CD56^+^/CD16^+^ | Anti-CD3-PerCP,  Anti-CD56-PE,  Anti-CD16-PE |
| 8 | γδ T lymphocytes | CD3^+^TCR- γδ ^+^ | Anti-CD3-PerCP  Anti-TCR- γδ -FITC |

FITC = fluorescein isothiocyanate

PE = phycoerythrin

PerCP = peridinin chrolophyll protein

APC = allophycocyanin
